# Differences between Groups of Family Physicians with Different Attitudes towards At-Risk Drinkers: A Post Hoc Study of the ODHIN Survey in Portugal

**DOI:** 10.1155/2016/3635907

**Published:** 2016-01-17

**Authors:** Frederico Rosário, Marcin Wojnar, Cristina Ribeiro

**Affiliations:** ^1^Preventive Medicine Institute, Faculty of Medicine of Lisbon, Edifício Egas Moniz, Avenida Professor Egas Moniz, 1649-028 Lisbon, Portugal; ^2^Tomaz Ribeiro's Primary Health Care Center, Avenida 25 de Abril, 3460-541 Tondela, Portugal; ^3^Department of Psychiatry, Medical University of Warsaw, Nowowiejska 27, 00-665 Warsaw, Poland; ^4^Department of Psychiatry, University of Michigan, Ann Arbor, MI 48105, USA

## Abstract

*Introduction*. We have recently shown that family physicians can be classified into two groups based on their attitudes towards at-risk drinkers: one with better and the other with worse attitudes.* Objective*. To compare the two groups regarding demographics, alcohol-related clinical practice, knowledge of sensible drinking limits, and barriers and facilitators to working with at-risk drinkers.* Methods*. A random sample of 234 Portuguese family physicians who answered the Optimizing Delivery of Health Care Interventions survey was included. The questionnaire asked questions on demographics, alcohol-related clinical practice, knowledge of sensible drinking limits, and barriers and facilitators to working with at-risk drinkers.* Results*. Family physicians with better attitudes were younger (*p* = 0.005) and less experienced (*p* = 0.04) and with higher male proportion (*p* = 0.01). This group had more hours of postgraduate training (*p* < 0.001), felt more prepared to counsel risky drinkers (*p* < 0.001), and considered themselves to have better counselling efficacy (*p* < 0.001). More family physicians in the group with worse attitudes considered that doctors cannot identify risky drinkers without symptoms (*p* = 0.01) and believed counselling is difficult (*p* = 0.005).* Conclusions*. Family physicians with better attitudes had more education on alcohol and fewer barriers to work with at-risk drinkers. These differences should be taken into account when designing implementation programs seeking to increase alcohol screening and brief advice.

## 1. Introduction

A significant proportion of patients seen by family physicians drink alcoholic beverages above recommended limits [1–3], putting them at risk of developing alcohol-related diseases [4]. Family physicians stand as the ideal health professionals to identify and advise patients to cut down on their drinking [5], and the majority of them declare their support for alcohol screening and advice [6, 7]. However, most family physicians remain unwilling to implement alcohol screening and brief interventions in routine clinical practice [5].

Several studies dwelled on the reasons why such contradiction exists [8–15]. They came out with a vast number of barriers standing between physicians' support for early intervention for alcohol problems and their uptake of these practices. These barriers go from environmental constraints (lack of time, lack of counselling materials, and lack of support) to physician-related limitations (lack of training, fear to antagonize the patient, and physicians' own attitudes towards at-risk drinkers). It seems clear that only broadband implementation programs covering all these dimensions can successfully help family physicians jump over all these hurdles.

Physicians' attitudes towards excessive drinkers are a key aspect to have into consideration when designing alcohol screening and brief interventions implementation programs. A previous study showed that training and support increased physicians' intervention rates but only of those who already felt secured and committed in working with risky drinkers; those feeling insecure and uncommitted in the first place worsened their attitudes [10]. These findings suggest the existence of distinct attitude-based family physicians groups with specific training and support needs. The identity of these groups remained elusive up until recently, when we were able to identify them in a sample of Portuguese family physicians [16]. Briefly, we measured Portuguese family physicians' attitudes towards risky drinkers with the Short Alcohol and Alcohol Problems Perception Questionnaire (SAAPPQ). SAAPPQ's scores were submitted to cluster analysis. With this analysis we were able to distinguish two different groups of Portuguese family physicians with unequal sizes: the first, comprising nearly 60% of the sample, formed by physicians with lower attitude scores towards at-risk drinkers; the second, comprising the remaining 40%, formed by physicians with higher attitude scores. We believe these findings will help to better design implementation programs by tailoring them to the emotional needs of physicians in each group.

This paper aims to characterize the above-mentioned groups by comparing their characteristics and views on barriers and facilitators for alcohol screening and brief advice. We hypothesize that family physicians with better attitudes towards at-risk drinkers report fewer constraints in working with them.

## 2. Methods

### 2.1. Sampling

A proportional random sampling strategy was conducted from April to June 2012. The Portuguese family physician national database, from which the sample was extracted, was stratified by age, sex, and health region. Selected family physicians were invited by e-mail to fill in the online questionnaire, available at a specifically designed and secured website. The survey was part of the Optimizing Delivery of Health Care Interventions (ODHIN) project. This was a four-year research project (2011–2014), cofinanced by the European Union, which included nine European countries. The project focused on the implementation of screening and brief intervention programs for hazardous and harmful drinking in primary health care. The survey instrument is available at the ODHIN project webpage [17].

A response rate of 30% was assumed based on previous studies showing that e-mailed surveys' response rates are usually low [18]. With this in mind, 850 family physicians were invited to participate in order to achieve the project's requested sample of 250 physicians. To increase participation rate, two e-mail reminders with a three-week interval were sent encouraging family physicians to fill in the survey.

### 2.2. Survey Instrument

The questionnaire was adapted from questionnaires applied in the World Health Organization Phase III strand I study [19] and in a primary care survey conducted in England [13]. The questionnaire asked family physicians to report on demographics; education and training on alcohol; what family physicians considered to be the upper limit for alcohol consumption before advising a healthy man or a nonpregnant healthy woman to reduce or stop drinking; alcohol-related clinical practice; attitudes towards risky drinkers, measured with the SAAPPQ (data on attitudes is reported elsewhere [20] and will not be described here); and barriers and facilitators for implementation of alcohol screening and brief advice.

### 2.3. Data Collection

Participants answered the survey through a secured website. They received an e-mail invitation explaining the study's objectives, survey filling details, and a direct website link. The data collection method was completely anonymous and did not retain any information that could be used to differentiate respondents from nonrespondents.

### 2.4. Data Management

Previous education and training on alcohol was dichotomized from a self-reported ordinal scale into “less than four hours” or “four or more hours” of alcohol specific education and training. Beliefs about family physicians' effectiveness after being adequately trained in reducing patients' alcohol consumption were dichotomized into “effective” or “ineffective.”

According to the Portuguese guidelines [21], upper limit of alcohol consumption was dichotomized as two standard drinks/day or any other answer for a healthy man and one standard drink/day or any other answer for a nonpregnant healthy woman.

Alcohol-related clinical practice questions were recoded from a self-reported ordinal scale as follows: asking patients about alcohol even if they do not was dichotomized into “All the time/Most of the time” or “Some of the time/Rarely or never”; obtaining information on patients drinking alcohol moderately was dichotomized into “Always/As indicated” or “Occasionally/Rarely or Never”; preparedness to counsel patients reducing alcohol consumption was dichotomized into “Very prepared/Prepared” or “Unprepared/Very unprepared”; effectiveness in reducing patients' alcohol consumption was dichotomized into “Very effective/effective” or “Ineffective/Very ineffective”; number of times a blood test was requested in the last year because of concern about alcohol consumption was dichotomized into “More than twelve times” or “Twelve times or less”; number of self-reported patients managed specifically for their hazardous drinking or alcohol-related problems in the last year was dichotomized into “Less than seven” or “Seven or more.”

Finally, barriers and facilitators were recoded as “Don't know/Not at all” or “Little/Quite a bit/Very much” to differentiate between physicians who expressed agreement with the statement and those in disagreement or who had no opinion.

### 2.5. Statistical Analysis

Data are shown as mean ± standard deviation or frequency distribution as appropriate. Family physicians groups were compared with independent samples *t*-test for continuous variables and chi-square or Fisher's exact test for categorical variables, as appropriate. A two-tailed *p* value <0.05 was considered for significance. Analysis was performed with R^©^ 3.0.2 (The R Foundation for Statistical Computing).

## 3. Results

### 3.1. Demographics

Sampled family physicians were on average 52.3 ± 8.7 years old and had 23.0 ± 9.4 years of experience working as family physicians, and the majority were female (*N* = 150, 64.1%). Almost all family physicians were working in an urban (*N* = 104, 44.5%) or mixed urban/rural (*N* = 96, 41.0%) practice; the remainder (*N* = 34, 14.5%) were working in a rural practice.

Family physicians with better attitudes towards at-risk drinkers were younger and less experienced and with higher proportion of male doctors than the group with worse attitudes (Table 1). The groups had similar practice distributions.

### 3.2. Education and Training on Alcohol

A majority of physicians (*N* = 141, 60.3%) reported having less than 4 hours of training on alcohol and alcohol-related problems. Almost all doctors (*N* = 220, 94.0%) believed that with adequate information and training family physicians would achieve higher effectiveness in helping patients to cut down on their drinking. Family physicians with better attitudes towards risky drinkers reported higher training in this specific area (Table 2). More doctors in this group also believed family physicians could be more effective with proper training.

### 3.3. Drinking Limits

Ninety-eight participants (41.9%) reported they would consider two standard drinks as the upper limit for alcohol consumption before they would advise a healthy adult man to cut down. A similar proportion (*N* = 102, 43.6%) answered one unit per day when asked the same question for a nonpregnant healthy woman.

We found no differences between the groups in respect to sensible drinking limits (Table 3).

### 3.4. Alcohol-Related Clinical Practice

Most family physicians (*N* = 178, 76.1%) indicated they ask patients frequently about alcohol even if patients do not ask about it. A majority also reported obtaining information on alcohol always or at least as indicated (*N* = 210, 89.7%); feeling prepared to counsel patients to cut down (*N* = 190, 81.2%); and feeling effective in helping patients to change their alcohol habits (*N* = 141, 60.3%). Nearly six out of ten family physicians (*N* = 138, 59.0%) said they have taken or requested a blood test more than 12 times in the last year because of concern about alcohol consumption, and 69.7% (*N* = 163) reported having managed in the last year at least 7 patients specifically for their hazardous drinking or alcohol-related problems.

Both groups gave similar answers concerning alcohol-related clinical practice except when it comes to feeling prepared to counsel, and effective in helping, patients to cut down on their drinking: more family physicians with better attitudes felt prepared and effective in doing so (Table 4).

### 3.5. Barriers to Alcohol Screening and Brief Advice

In general, nearly half or more participants agreed with all suggested barriers.

In respect to health provider-related barriers, family physicians agreed doctors believe counselling is too difficult (*N* = 212, 90.6%); are not trained in counselling for reducing alcohol consumption (*N* = 196, 83.8%); do not know how to identify problem drinkers who have no obvious symptoms of excess consumption (*N* = 173, 73.9%); feel awkward asking patients questions about alcohol (*N* = 172, 73.5%); may have alcohol problems (*N* = 161, 68.8%); have disease model training (*N* = 156, 66.6%); have a liberal attitude towards alcohol (*N* = 149, 63.7%); and think preventive health should be patients' responsibility and not theirs (*N* = 112, 47.9%).

Regarding patient-related barriers, family physicians agreed doctors believe patients would disregard their advice (*N* = 190, 81.2%) and they would resent being asked about alcohol (*N* = 134, 57.3%).

Concerning organizational barriers, family physicians agreed doctors lack suitable counselling materials available (*N* = 196, 83.8%); are too busy dealing with other patients' problems (*N* = 194, 82.9%); are not sufficiently encouraged by their contract to work with alcohol problems (*N* = 193, 82.5%); and lack a suitable screening device available (*N* = 184, 78.6%).

Family physicians from both groups overlapped their views on most suggested barriers (Table 5). Their opinions differed only on two health provider-related barriers since more family physicians from the worse attitudes group agreed doctors do not know how to identify problem drinkers who have no obvious symptoms of excess consumption (*p* = 0.01) and believe counselling is too difficult (*p* = 0.005). We also found a trend towards more family physicians from the worse attitudes group agreeing doctors feel awkward asking patients questions about alcohol (*p* = 0.07).

### 3.6. Facilitators of Alcohol Screening and Brief Advice

The vast majority agreed with all suggested incentives to implement alcohol screening and brief intervention.

In respect to health provider-related facilitators, family physicians agreed they would be encouraged to do more early intervention for hazardous alcohol consumption if early intervention for alcohol was proven to be successful (*N* = 226, 96.6%).

Concerning patient-related facilitators, family physicians agreed they would be encouraged to do more early interventions if patients requested health advice about alcohol consumption (*N* = 229, 97.9%) and if public health education campaigns in general made society more concerned about alcohol (*N* = 228, 97.4%).

As to organizational facilitators, participants agreed they would be encouraged to do more early interventions if general support services (self-help/counselling) were readily available to refer patients to (*N* = 229, 97.9%); quick and easy counselling materials were available (*N* = 228, 97.4%); training programs for early intervention were available (*N* = 226, 96.6%); quick and easy screening questionnaires were available (*N* = 222, 94.1%); and salary and working conditions were improved (*N* = 192, 82.1%).

Family physicians from both groups showed similar views on all suggested barriers (Table 6).

## 4. Discussion

This study shows that family physicians with better attitudes towards risky drinkers report fewer constraints to implement alcohol screening and brief advice, specifically when it comes to physician-related barriers. Both groups reported similar views on organizational and patient-related barriers and differed only in two physician-related barriers concerning beliefs about knowledge and skills fundamental to approach patients' alcohol-drinking habits. We also found a trend towards more doctors in the worse attitudes group feeling uncomfortable asking patients about alcohol. Taken together, these findings suggest that doctors with worse attitudes have higher knowledge and skills-training needs and also lower confidence levels in their abilities to implement alcohol screening and brief advice. This claim finds support in the differences found in education and training on alcohol: the group with better attitudes had more hours of postgraduate training, which may imply that previous training may have boosted physicians' knowledge, skills, and confidence; they also believed that family physicians can increase their counselling effectiveness if they receive proper training. However, this was a cross-sectional study, which means that causality cannot be inferred. It is possible that physicians already with better attitudes prior to training sought to obtain education on alcohol simply because they had interest in alcohol issues. On the other hand, having more education and training on alcohol does not seem to improve knowledge of daily drinking limits, which points to the need of improving the way information is delivered during training.

Despite the differences found on the above-mentioned barriers, the groups shared similar views on all suggested facilitators. It seems that family physicians in both groups can equally benefit from changes in the primary care infrastructure. Possible changes are the availability of screening and counselling materials (e.g., having a screening tool installed on the electronic health record software, leaflets to hand over to patients), easy access to support services (e.g., specialist advice on difficult cases, a working referral network), and better payment and working conditions overall. Social pressure may also play an important part in increasing alcohol consumption discussions as most physicians would like to see patients asking for advice on this specific issue, pointing public health education campaigns as a possible way to achieve this.

Other interesting results relate to clinical practice issues. When advising patients to cut down, more family physicians with better attitudes reported feeling prepared and effective in reducing alcohol consumption. Despite this, we found similar self-reported practice behaviours on the number of patients advised, blood tests required, and information obtained on alcohol from patients. It seems that having more positive feelings towards at-risk drinkers does not necessarily translate into more self-reported screening and advice. This suggests that, despite its importance, addressing only physicians' emotional aspects may fail to significantly increase screening and advice rates.

Groups differed also in demographic variables. Younger, less experienced family physicians reported better attitudes towards patients with excessive alcohol consumption. When it comes to gender, male physicians reported feeling more role-secured and therapeutically committed towards working with at-risk drinkers than female doctors. How to interpret these results remains elusive.

### 4.1. Comparison with Previous Research

Physicians' agreement with barriers and facilitators found in this study mirrors that reported in the literature. Many studies point to organizational factors as a major impediment to implement screening and brief interventions. The most common organizational barriers cited in these studies are lack of time [6, 9, 12, 22–25]; lack of screening tools [9, 12]; lack of counselling materials [9, 12]; and lack of support [6, 7, 24]. Evidence also underlines similar patient- and physician-related factors as important barriers. Patient-related barriers most often reported relate to fear of upsetting patients [5, 6, 15] and belief that patients will disregard advice to cut down [5, 12, 22]. As to physician-related barriers, doctors often report lack of training [5, 6, 9, 15, 22]; lack of knowledge and skills, [6, 9, 15]; and low confidence and motivation to identify risky drinkers and deliver advice [9, 15]. Literature also shows physicians agree that tackling these organizational barriers would facilitate implementation [6, 12]. These similarities strengthen the reliability of the results found in our study.

### 4.2. Implications for Implementation Research

Based on the findings of this study it seems reasonable to postulate that differences between groups relate essentially to their views on alcohol issues and to the way they feel about addressing those issues with patients. As such, we hypothesize that fine-tuning implementation programs only to the differences found may set the ground to an improvement in the way physicians think and feel about alcohol-related problems but will probably fail to achieve higher screening and advice rates. We believe we need a more comprehensive strategy to address the way family physicians deal with these issues in their daily practice. For example, we must carefully consider the role of other primary health care professionals. Nurses doing screening and even delivering brief advice might have a positive impact on family physicians own screening and advice rates. Receptionists handing self-administered screening tools to patients might boost screening rates. Including residents in the program may also be a positive influence. Implementation programs must be carefully planned if one wants to change deeply rooted routine clinical practice, which usually obliviates alcohol screening and brief advice.

### 4.3. Limitations

The results of this study must be interpreted having its limitations in mind. The first is the low response rate achieved. Electronic surveys usually result in low response rates, but they seem to allow for generalization when the sampling method is conducted using probability samples of full populations [18]. However, we cannot be certain the sample represents the views of all Portuguese family physicians.

As mentioned earlier, this was a cross-sectional study, which does not allow establishing causality paths. The example given earlier is illustrative: we cannot ascertain the direction of the association between training and physicians' attitudes. It is possible that training may have improved physicians' attitudes but is also conceivable that physicians with better attitudes to begin with sought to get training on alcohol-related problems. Nevertheless, results are consistent with similar studies previously reported, which gives support to the conclusions drawn.

Finally, data are self-reported and no external data validation was conducted. Some variables such as number of patients advised on alcohol, number of blood tests required, or frequency of asking about alcohol consumption are personal estimations and possibly subjected to bias.

## 5. Conclusions

Family physicians with better attitudes towards problem drinkers report fewer physician-related barriers to implement alcohol screening and brief interventions. They face similar difficulties concerning organizational and patient-related barriers and also enablers of these practices. We plan to integrate these results in the design of a new implementation program for alcohol problems in Portugal, seeking to increase family physicians' screening and brief advice.

## Figures and Tables

**Table 1:** Demographic characteristics of the sample of Portuguese family physicians participating in the survey.

Demographics	Group with worse attitudes	Group with better attitudes	*p*
Age	53.7 ± 7.7	50.3 ± 9.8	0.005^a^
Years practicing as a family physician	24.0 ± 8.6	21.4 ± 10.3	0.04^a^
Sex *N* (%)			
Male	41 (29.3)	43 (45.7)	0.01^b^
Female	99 (70.7)	51 (54.3)	
Practice characteristic *N* (%)			
Urban	62 (44.3)	42 (44.7)	0.57^b^
Rural	23 (16.4)	11 (11.7)	
Mixed urban/rural	55 (39.3)	41 (42.7)	

^a^Independent samples *t*-test; ^b^chi-square test.

**Table 2:** Number of hours of training on alcohol received and views on effectiveness in reducing patients' alcohol consumption if properly trained.

Training	Group with worse attitudes *N* (%)	Group with better attitudes *N* (%)	*p* ^a^
Hours of any form of postgraduate training on alcohol ever received			
<4 hours	98 (70.0)	43 (45.7)	<0.001
≥4 hours	42 (30.0)	51 (54.3)	
Would family physicians be effective with adequate information and training?			
Effective	128 (91.4)	92 (97.9)	0.04
Ineffective	12 (8.6)	2 (2.1)	

^a^Chi-square test.

**Table 3:** Family physicians' knowledge about sensible drinking limits.

Sensible drinking limits	Group with worse attitudes *N* (%)	Group with better attitudes *N* (%)	*p* ^a^
Upper daily limit for a healthy man			
=2 standard drinks/units per day	57 (40.7)	41 (43.6)	0.66
≠2 standard drinks/units per day	83 (59.3)	53 (56.4)	
Upper daily limit for a nonpregnant healthy woman			
=1 standard drink/unit per day	62 (44.3)	40 (42.6)	0.79
≠1 standard drink/unit per day	78 (55.7)	54 (57.4)	

^a^Chi-square test.

**Table 4:** Alcohol-related clinical practice behaviours.

	Group with worse attitudes *N* (%)	Group with better attitudes *N* (%)	*p* ^a^
Ask about alcohol even if patients do not			
All the time/Most of the time	102 (72.9)	76 (80.9)	0.16
Some of the time/Rarely or never	38 (27.1)	18 (18.9)	
Extent to which information was obtained on patients' drinking alcohol moderately			
Always/As indicated	124 (88.6)	86 (91.5)	0.47
Occasionally/Rarely or Never	16 (11.4)	8 (8.5)	
Feel prepared to counsel patients reducing alcohol consumption			
Very prepared/Prepared	104 (74.3)	86 (91.5)	<0.001
Unprepared/Very unprepared	36 (25.7)	8 (8.5)	
Feel effective in helping patients reducing alcohol consumption			
Very effective/effective	68 (48.6)	73 (77.7)	<0.001
Ineffective/Very ineffective	72 (51.4)	21 (22.3)	
Number of times a blood test was requested in the last year because of alcohol concern			
>12 times	77 (55.0)	61 (64.9)	0.13
≤12 times	63 (45.0)	33 (35.1)	
Number of patients managed for alcohol in the last year			
≥7 patients	92 (65.7)	71 (75.5)	0.11
<7 patients	48 (34.3)	23 (24.5)	

^a^Chi-square test.

**Table 5:** Agreement with selected barriers for the implementation of alcohol screening and brief interventions.

Barriers	Group with worse attitudes *N* (%)	Group with better attitudes *N* (%)	*p* ^a^
Doctors are too busy dealing with other problems	120 (85.7)	74 (78.7)	0.16
Doctors have a disease model training and do not think about prevention	99 (70.7)	57 (60.6)	0.11
Doctors think preventive health should be patients' responsibility not theirs	71 (50.7)	41 (43.6)	0.29
Doctors are not sufficiently encouraged to work with alcohol problems	111 (79.3)	82 (87.2)	0.12
Doctors feel awkward about asking questions about alcohol consumption	109 (77.9)	63 (67.0)	0.07
Doctors do not know how to identify problem drinkers who have no obvious symptoms	112 (80.0)	61 (64.9)	0.01
Doctors do not have a suitable screening device to identify problem drinkers	115 (82.1)	69 (73.4)	0.11
Doctors do not have suitable counselling materials available	117 (83.6)	79 (84.0)	0.92
Doctors are not trained in counselling for reducing alcohol consumption	124 (88.6)	78 (83.0)	0.22
Doctors believe that alcohol counselling is too difficult	133 (95.0)	79 (84.0)	0.005
Doctors do not believe that patients would take their advice	117 (83.6)	73 (77.7)	0.26
Doctors themselves have a liberal attitude towards alcohol	91 (65.0)	58 (61.7)	0.61
Doctors themselves may have alcohol problems	96 (68.6)	65 (69.1)	0.93
Doctors believe that patients would resent being asked about their alcohol consumption	82 (58.6)	52 (55.3)	0.62

^a^Chi-square test.

**Table 6:** Agreement with selected facilitators for the implementation of alcohol screening and brief interventions.

Facilitators	Group with worse attitudes *N* (%)	Group with better attitudes *N* (%)	*p*
Public health education campaigns	136 (97.1)	92 (97.9)	1.0^a^
Patients requesting advice about alcohol	139 (99.3)	90 (95.7)	0.16^a^
Having quick and easy screening questionnaires	134 (95.7)	88 (93.6)	0.55^a^
Having quick and easy counselling materials	136 (97.1)	92 (97.9)	1.0^a^
Proof of alcohol's early intervention effectiveness	136 (97.1)	90 (95.7)	0.72^a^
Training programs for early intervention for alcohol	136 (97.1)	90 (95.7)	0.72^a^
General support services (self-help/counselling)	137 (97.9)	92 (97.9)	1.0^a^
Better salary and working conditions	115 (82.1)	77 (81.9)	0.96^b^

^a^Fisher's exact test; ^b^chi-square test.
